# Recurrence quantification analysis of heart rate variability to detect both ventilatory thresholds

**DOI:** 10.1371/journal.pone.0249504

**Published:** 2021-10-07

**Authors:** Giovanna Zimatore, Lavinia Falcioni, Maria Chiara Gallotta, Valerio Bonavolontà, Matteo Campanella, Marco De Spirito, Laura Guidetti, Carlo Baldari

**Affiliations:** 1 Department of Theoretical and Applied Sciences, eCampus University, Novedrate (CO), Italy; 2 IMM-CNR, Bologna, Italy; 3 Department of Movement, Human and Health Sciences, University of Rome “Foro Italico”, Rome, Italy; 4 Department of Physiology and Pharmacology "Vittorio Erspamer", Sapienza University of Rome, Rome, Italy; 5 Department of Basic Medical Sciences, Neuroscience and Sense Organs, University “Aldo Moro”, Bari, Italy; 6 Fondazione Policlinico Universitario A. Gemelli IRCCS, Università Cattolica del Sacro Cuore Rome, Rome, Italy; 7 Niccolò Cusano University, Rome, Italy; University of Bourgogne France Comté, FRANCE

## Abstract

Aims of this study were: to verify if Recurrence Quantification Analysis (RQA) of Heart Rate Variability (HRV) time series could determine both ventilatory thresholds in individuals with different fitness levels, and to assess the validity of RQA method compared to gas-exchange method (GE). The two thresholds were estimated in thirty young individuals during incremental exercise on cycle-ergometer: Heart rate (HR), Oxygen consumption (VO_2_) and Workload were measured by the two methods (RQA and GE). Repeated measures ANOVA was used to assess main effects of methods and methods-by-groups interaction effects for HR, VO_2_ and Workload at aerobic (AerT) and anaerobic (AnT) thresholds. Validity of RQA at both thresholds was assessed for HR, VO_2_ and Workload by Ordinary Least Products (OLP) regression, Typical Percentage Error (TE), Intraclass Correlation Coefficients (ICC) and the Bland Altman plots. No methods-by-groups interaction effects were detected for HR, VO_2_ and Workload at AerT and AnT. The OLP analysis showed that at both thresholds RQA and GE methods had very strong correlations (*r* >0.8) in all variables (HR, VO_2_ and Workload). Slope and intercept values always included the 1 and the 0, respectively. At AerT the TE ranged from 4.02% (5.48 bpm) to 10.47% (8.53 Watts) (HR and Workload, respectively) and in all variables ICC values were excellent (≥0.85). At AnT the TE ranged from 2.53% (3.98 bpm) to 6.64% (7.81 Watts) (HR and Workload, respectively) and in all variables ICC values were excellent (≥0.90). Therefore, RQA of HRV time series is a new valid approach to determine both ventilatory thresholds in individuals with different physical fitness levels, it can be used when gas analysis is not possible or not convenient.

## Introduction

In exercise physiology the “threshold “concept is frequently used to assess both aerobic fitness and performance. The gas exchange (GE) method allows detecting ventilatory and gas exchange response and it is highly utilized for threshold identification [1, 2]. Although the GE method represents the gold standard, more sustainable and inexpensive approaches can be easily applied to determine the thresholds when gas analysis is non convenient or not possible.

In a previous work we proposed a new non-linear method based on Recurrence Quantification Analysis (RQA) of Heart Rate Variability (HRV) time series to estimate the aerobic threshold (AerT) in obese subjects [3]. In this special population the AerT (or first lactate threshold according to Binder and colleagues [2]), represents a useful parameter to identify the most appropriate physical exercise intensity in order to reduce body weight (BW) and to improve physical fitness [4]. With increasing exercise intensity above the AerT, the lactate production rate becomes higher than the metabolizing capacity in the working muscle. This leads to an increased production of carbon dioxide and to a steeper increase of ventilation, while the increase of oxygen consumption continues to rise linearly with increasing workload. This onset of exercise-induced hyperventilation (i.e.an overproportioned increase ventilation in relation to carbon dioxide production) represents the Anaerobic threshold (AnT) or second lactate threshold [1, 2]. Above the AnT the muscular lactate production rate exceeds the systemic lactate elimination rate. Therefore, this phase is represented by a more pronounced ventilation and a further increase in carbon dioxide production in an attempt to compensate for the marked rise in lactate [1, 2, 5].

The AerT and AnT are not always clearly distinguishable in subjects with low level of fitness such as obese individuals. This population cannot perform an incremental physical exercise for enough long time and consequently usually the AerT is usually the only ventilatory parameter that can be determined [6]. On the other side, the AerT and AnT can be more easily observed in a healthy population with a higher fitness level as athletes. Therefore, this population should be considered for developing a new method to estimate both ventilatory thresholds. Previous studies in athletes proposed several methods to provide information about the relationship between heart rate and the AerT and AnT [7–12]. RQA can be defined as a graphical, statistical and analytical tool used by several disciplines from physiology [13–15], to earth science [16] and economics [17, 18] to detect phase transitions. The method based on RQA of HRV time series to distinguish the two ventilatory thresholds still needs to be established. Nowadays it is extremely easy to monitor the heart rate using low-cost, non-invasive, and mobile systems [19–21]. Since the use of these devices does not necessarily require the presence of the subject in the laboratory, from a logistic point of view, the RQA of HRV time series could be an extremely useful method for assessing sport performance and for planning training intensity in athletes in situations where gas analysis is not permitted.

The purpose of this study was to verify if the RQA of HRV time series proposed for obese individuals can be applied also to healthy young subjects. In particular, the first aim was to assess if the new non-linear method based on RQA could detect both ventilatory thresholds in individuals with different physical fitness levels. The second aim of the study was to investigate the validity of the RQA method compared to the GE method in thresholds detection.

## Materials and methods

In this paper the two evaluation methods were called Gas Exchange method (GE) and Recurrence Quantification Analysis method (RQA). In addition, the two thresholds, Aerobic (AerT) and Anaerobic (AnT), were called Aerobic Gas Exchange Threshold (AerT_GE_), Anaerobic Gas Exchange Threshold (AnT_GE_), Aerobic Recurrence Quantification Analysis Threshold (AerT_RQA_), and Anaerobic Recurrence Quantification Analysis Threshold (AnT_RQA_).

### Participants

A priori power analysis indicated that a total sample size of 30 subjects were required to detect a medium effect size (f = 0.25) given a coefficient of correlation p = 0.60 with 80% power and alfa = 0.05, using ANOVA repeated measure, within factors.

Thirty subjects (2 females; 28 males) (age = 15.7 ± 2.7 years) were recruited for this study. Participants were divided in three groups: competitive rowers (group A, n = 8), recreational rowers (group B, n = 8) and other recreational sports (group C, n = 14). The A group was made up of competitive rowers, i.e. athletes who performed a minimum of 5 workouts per week and have participated in a regional and/or national competitions in the previous year. The subjects of group B attended rowing activities twice a week. Both the A and B groups were recruited from the “Circolo Canottieri Tirrenia Todaro” of Rome. The C group was made up of subjects who practice recreational activities different to rowing (twice a week).

All participants underwent clinical examinations to exclude any side effects to physical activity. However, a medical certificate for competitive sport activities or noncompetitive sport activities was requested. Moreover, subjects of all groups had the same main anthropometric characteristics except height (see Table 1). The exclusion criteria were neuropathy, autonomic dysfunction, cardiovascular diseases. All subjects (or their parents if <18 years) provided a written informed consent before the beginning of the study. This study was conducted in accordance with the Declaration of Helsinki and approved by the CAR-IRB—University of Rome “Foro Italico” Committee (Approval N° CAR 37/2020).

**Table 1:** Characteristics of the study sample (mean value ± SD).

Parameters	All (n = 30)			A (n = 8)			B (n = 8)			C (n = 14)		
Age (years)	15.7	±	2.7	17.13	±	4.22	16.0	±	2.0	14.3	±	1.6
Height (cm)	176.6	±	8.9	183.85	±	8.05*	174.6	±	6.7	174.3	±	8.9
Weight (kg)	70.2	±	13.9	73.5	±	7.7	69.9	±	11.7	68.6	±	17.8
BMI (kg/m^2^)	22.3	±	3.4	21.7	±	1.3	22.9	±	3.5	22.3	±	4.2
FM%	20.9	±	5.9	17.6	±	3.1	21.0	±	6.5	22.8	±	6.2

*p = 0.039 A vs C.

### Procedures

After clinical examination and anthropometric measurements, all subjects performed a graded incremental exercise test on cycle-ergometer. All subjects were tested in the morning (between 9:00 and 12:00 a.m.), and under similar environmental conditions (temperature 21–22˚ C; humidity 50–60%). Subjects had their usual breakfast at least 90 minutes before the test session. All tests were performed at the Department of Movement, Human and Health Sciences at the University of Rome “Foro Italico”.

### Anthropometric measurements

The following participants’ anthropometric measurements were assessed: body-weight, height, Body Mass Index (BMI) and percent of fat mass (FM%). Weight and height were measured using a scale and a stadiometer to the nearest 0.1 kg and 0.1 cm, respectively. BMI was calculated as the ratio between weight in kg and the square of height in meters (kg/m^2^). FM% was measured by bioelectrical impedance method (BIA AKERN 101 Anniversary, Pontassieve, FI, Italy).

### Incremental exercise test

The incremental exercise test on the cycle-ergometer was performed with the following protocol: participants started with a 1 minute rest period sitting on the bike, followed by 1 minute of unloaded pedaling (0 Watt). The workload was then increased by 20 Watts/minute for the A group (protocol 1) and by 15 Watts/minute for the B and C group (protocol 2), in order to maintain the total exercise time within about 15 minutes. Participants were asked to keep a cadence of 60–70 revolutions per minute (rpm). During the test, perception of physical exertion was assessed using OMNI Scale of Perceived Exertion (0–10 scale, [22]) 15 seconds prior to the end of every stage. The participants were asked to rate on a scale from 0 (extremely easy) to 10 (extremely hard) their subjective intensity of effort. The test ended when one of the following conditions was reached: a value of 10 on OMNI Scale of Perceived Exertion, the 90% of the subject’s predicted HRmax (beats/min) or a respiratory exchange ratio equal to 1.1.

The HR was recorded by a chest belt (HRM-Dual™, Garmin®) contemporarily to Oxygen consumption (VO_2_, mL/min), carbon dioxide production (VCO_2_, mL/min), and pulmonary ventilation (VE, mL/min) that were measured by an automatic gas analyzer (Quark RMR-CPET Cosmed™, Rome, Italy) [23] (See S2 Table).

### Detection of the AerT and AnT (GE method)

Gas exchange method (GE) is the gold standard to detect both AerT and AnT as reported by Meyer et al. (2005) [1]. The AerT_GE_ (that occurred at time named T_1_), was determined offline for each subject by plotting the ventilatory equivalent of oxygen (VE/VO_2_) as a function of VO_2_ to identify the point where the VE/VO_2_ reached its lowest value during the exercise test [24–26]. This procedure was confirmed by a graph with VCO_2_ on the y axis and VO_2_ on the x axis: two regression lines are fitted for the upper and the lower part of the relation and their intersection represents the AerT_GE_ (V-slope method, [1]).

Similarly, the AnT_GE_ (that occurred at time named T_2_) was determined by plotting the ventilatory equivalent of carbon dioxide (VE/VCO_2_) as a function of VO_2_ to identify the point where the VE/VCO_2_ reached its lowest value during the incremental test. This procedure was confirmed by a graph with VE on the y axis and VCO_2_ on the x axis: two regression lines are fitted for the upper and the lower part of the relation and their intersection represents the AnT_GE_ [1].

HR e VO_2_ values corresponding at the two thresholds were reckoned as mean values of the last 30 seconds of the workload.

### Recurrence Quantification Analysis method (RQA)

RQA-based method is widely explained in a previous paper [3]: it was observed that by recurrence analysis, in epoch-by-epoch (with sliding windows) mode (RQE), the rapid shifts from high to low (and vice versa) of percent of determinism (DET, the percentage of recurrence points which form diagonal lines) is usually an indicator of regime changes and phase transitions [27, 28]. In fact, DET (High DET means high autocorrelation) is a universal marker of crisis in fields ranging from physiology to finance [29]. Laminarity (LAM, the percentage of recurrence points which form vertical lines), analogous to DET, measures the number of recurrence points which form vertical lines and indicates the amount of laminar phases (intermittency) in the system studied. In the present work we use both DET and LAM to study the increase in correlation and to identify the ventilatory thresholds; the present study was focused on chaos-order transitions and physical fatigue was considered as an order parameter acting at physiological level. More details can be found at http://www.recurrence-plot.tk/ and in [28, 30–32].

### Data preprocessing

As previously explained by Zimatore et al. (2020) [3], since the heart rate (HR) was continuously recorded and collected breath-by-breath, the time series of RR interval (temporal variation between the sequences of consecutive heartbeats) was reckoned by the ratio 60000/HR. Moreover, since HR physiologically increases in relation to the workload, this physiological trend was removed in order to analyze the cardiac regime changes (detrended RR interval) for each subject (the theoretical value of best linear fit on original time series were subtracted to every point belong to original time series). The optimization procedure of input parameters was discussed in a previous work [3], where the following same values were used:

- delay (lag) is set to 1,
- embedding dimension is set to 7,
- cut-off distance (radius) to 50% of mean distance between all pairs of points in time,
- line (minimal number of consecutive recurrences to score a determinism line) set to 4.

RQA is computed across consecutive distance matrices corresponding to consecutive and overlapping sliding windows (epochs) along the series, and this mode of analysis is called RQE (Recurrence Quantification by Epochs). The occurrence of an abrupt change of DET and LAM in adjacent windows corresponds to a transition in the dynamical regime of the signal. RQE analysis was carried out by adopting the same parameters setting as for the global mode (in the present study, when an epoch coincides with all points of the time series, the result is called RQA), plus the definition of windows having a length of 100 points (1 point correspond to 1 breath) and shifting of 1 point between consecutive windows, respectively.

RQA software, RQE.exe and RQC.exe version 8.1, are those included in RQA 14.1 [30]. RQE on sliding windows partially overlapped are carried on a detrended RR interval and then RQA measures are obtained. The software used for the RQA analysis, that generates Recurrence plots (RP) and other RQA utilities, is available from http://cwebber.sites.luc.edu.

### Detection of the AerT and AnT (RQA method)

**Fig 1:**
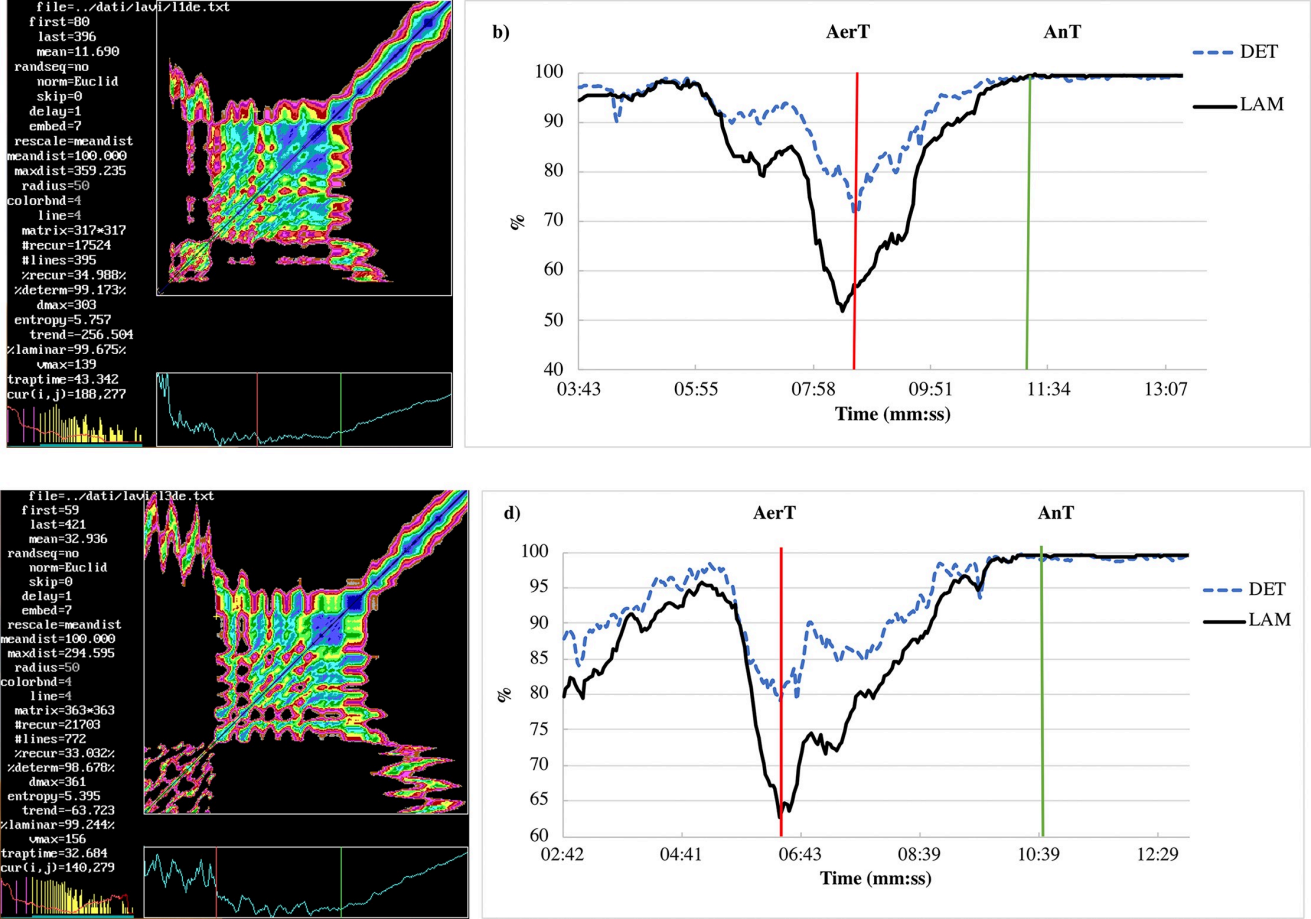
RP and RQE comparison. A comparison among two representative RP is shown in panel a (subject #1) and panel c (subject #3). Panel b (subject #1) and panel d (subject #3) show the percentage of recurrence points which form diagonal lines (DET) and the percentage of recurrence points which form vertical lines (LAM) epoch by epoch vs time (min:sec) of detrended RR interval. The red line enlightens the statistically relevant minimum that corresponds to the AerT and the green line corresponds to the AnT where the percent of determinism reaches saturation. Subject #1 is a competitive rower (group A, protocol 1) and subject #3 is a recreational rower (group B, protocol 2). The test starts at 00:00; AerT occurred at time T_1_, 8:38 and 5:49 for #1 and #3, respectively; AnT occurred at time T_2_, 11:24 and 11:01 for #1 and #3, respectively. A square pattern in the middle is easily distinguishable, the vertical red line is manually placed nearby before the first ventilatory transition, the green one nearby before the second one. The colors of RP reflect different Euclidean distances between trajectories and are analogous to geographical relief maps going from blue to red at increasing Euclidean distance. Distances greater than cut-off correspond to black regions.

The method of threshold detection based on RQA of RR intervals (HRV time series) can be described as follows: specifically, AerT_RQA_ was determined by the time point when the statistically relevant minimum of percent of determinism and laminarity occurred. AnT_RQA_, instead, occurred when percent of determinism is saturated (the saturation occur when DET was above 90% and the maximum value did not change for at least 20 seconds); both threshold time points correspond to evident changes in RP texture (see Fig 1). HR e VO_2_ values corresponding to the two thresholds were reckoned as mean values on the last 10 values of the original time series before the value corresponding to the two threshold times (T_1_ and T_2_).

### Statistical analysis

Repeated measures analysis of variance (RM ANOVA) was used to assess main effects of methods (RQA vs GE) and methods-by-groups (A, B and C) interaction effects for HR, VO_2_ and Workload at the AerT and the AnT. Agreements between RQA and GE methods at the AerT and the AnT were assessed for HR, VO_2_ and Workload by Ordinary Least Products (OLP) regression analysis [33]. Coefficients of determination (R^2^) and regression parameters (slope and intercept) with the 95% of confidence intervals (95% CI) were calculated for the OLP regression equation to determine fixed and proportional biases. The hypothesis of proportional and fixed bias was rejected when the 95% CI contained the value 1 for the slope and the 0 for the intercept, respectively. Percentage differences between the RQA and GE methods at the AerT and the AnT (reported as mean and range values) were calculated for HR, VO_2_ and Workload. RQA method validity was also assessed by comparing HR, VO_2_ and Workload at the AerT and the AnT vs the same variables measured by the GE method with a paired samples t-test. Measurement error was expressed in Typical percentage Error (TE) which was calculated by dividing the standard deviation of the difference percentage by $\sqrt{2}$. Intraclass correlation coefficients (ICC) was used as a parameter for criterion validity of the RQA method compared to the GE method at the AerT and the AnT for all variables (HR, VO_2_ and Workload).

The magnitude of the differences between RQA and GE methods was assessed using effect size statistics (as Cohen’s d) with 90% confidence interval (CI) and percentage change [34]. The effect size (d) was classified as follows: <0.2 = trivial, 0.2–0.6 = small, 0.6–1.2 = moderate, 1.2–2.0 = large and >2.0 = very large [35].

Bland-Altman plots were applied to determine the 95% limits of agreement (LoA) between the RQA and GE methods at the AerT and the AnT for the HR, VO2 and Workload [36].

Statistical significance was defined as p ≤0.05. All statistical analysis was performed by SPSS version 24.0 software (SPSS Inc., Chicago, IL).

## Results

In Table 2 all the main parameters at the two thresholds and at the peak exercise for each group, are reported.

**Table 2:** Main parameters for group (Mean value ± SD).

	Competitive Rowers (A)	Recreational Rowers (B)	Other Recreational Sports (C)
HR at AerT_GE_ (bpm·min^-1^)	150.0 ± 7.1	134.3 ± 14.3	132.8 ± 16.0
VO_2_ at AerT_GE_ (mL·min^-1^)	2409 ± 499	1662 ± 260	1637 ± 276
VO_2/BW_ at AerT_GE_ (mL·min^-1^·kg^-1^)	32.6 ± 4.0	24.1 ± 3.1	25.4 ± 7.6
RER at AerT_GE_	0.92 ± 0.07	0.91 ± 0.04	0.89 ± 0.04
Power at AerT_GE_ (Watt)	140 ± 41	83 ± 20	73 ± 18
HR at AerT_RQA_ (bpm·min^-1^)	154.5 ± 5.9	136.2 ± 11.0	137.0 ± 17.3
VO_2_ at AerT_RQA_ (mL·min^-1^)	2552 ± 524	1722 ± 239	1727 ± 330
VO_2/BW_ at AerT_RQA_ (mL·min^-1^·kg^-1^)	34.6 ± 4.5	25.1 ± 4.6	26.7 ± 8.1
RER at AerT_RQA_	0.93 ± 0.08	0.92 ± 0.05	0.92 ± 0.05
Power at AerT_RQA_ (Watt)	148 ± 40	81 ± 16	78 ± 20
HR at AnT_GE_ (bpm·min^-1^)	173.4 ± 4.6	164.4 ± 9.1	157.7 ± 14.3
VO_2_ at AnT_GE_ (mL·min^-1^)	3244 ± 670	2301 ± 268	2146 ± 376
VO_2/BW_ at AnT_GE_ (mL·min^-1^·kg^-1^)	43.9 ± 5.8	33.5 ± 5.4	33.2 ± 9.4
RER at AnT_GE_	1.02 ± 0.06	1.00 ± 0.03	0.99 ± 0.06
Power at AnT_GE_ (Watt)	205 ± 48	131 ± 17	119 ± 24
HR at AnT_RQA_ (bpm·min^-1^)	172.6 ± 5.8	163.4 ± 8.3	157.8 ± 14.0
VO_2_ at AnT_RQA_ (mL·min^-1^)	3228 ± 639	2300 ± 315	2120 ± 307
VO_2/BW_ at AnT_RQA_ (mL·min^-1^·kg^-1^)	43.7 ± 5.1	33.3 ± 4.2	32.8 ± 8.7
RER at AnT_RQA_	1.00 ± 0.07	1.01 ± 0.04	0.98 ± 0.05
Power at AnT_RQA_ (Watt)	205 ± 44	135 ± 16	122 ± 21
HR at Peak (bpm·min^-1^)	187.6 ± 5.0	183.0 ± 7.3	175.4 ± 15.1
VO_2_ at Peak (mL·min^-1^)	4029 ± 827	2930 ± 322	2615 ± 489
VO_2/BW_ at Peak (mL·min^-1^·kg^-1^)	54.6 ± 7.3	42.5 ± 4.2	40.2 ± 10.6
RER at Peak	1.12 ± 0.06	1.11 ± 0.03	1.07 ± 0.05
Power at Peak (Watt)	268 ± 68	174 ± 24	150 ± 31

HR (heart rate), VO_2_ (oxygen uptake expressed as ml/min), VO_2/BW_ (oxygen uptake expressed as ml/kg/min), power and RER (respiratory exchange ratio) at the AerT, AnT and peak of exercise using RQA and GE methods for each group.

Workload at the AerT and HR, VO_2_, and Workload at the AnT were not statistically different between RQA and GE methods. Significant differences were found between the two methods for HR at the AerT (HR at the AerT_GE_ = 137.77 ± 15.25 beats/min; HR at the AerT_RQA_ = 141.44 ± 15.35 beats/min; p = 0.026 d = 0.24, *small*) and for VO_2_ at the AerT (VO_2_ at the AerT_GE_ = 1849.56 ± 477.68 mL/min; VO_2_ at the AerT_RQA_ = 1945.37 ± 516.91 mL/min; p = 0.020 d = 0.19, *trivial*). No methods-by-groups interaction effects were detected for HR, VO_2_ and Workload at the AerT and the AnT as reported in Table 3).

**Table 3:** Main effects of methods (RQA vs GE) with Effect Size (Cohen’s d) and method-by-group (A, B and C) interaction effects (RM ANOVA).

		AerT		AnT		
	Method	Effect Size	Method-by-group	Method	Effect Size	Method-by-group
	p	d	p	p	d	p
**HR (beats/min)**	0.026*	0.24	0.766	0.616	0.05	0.907
**VO2 (mL/min)**	0.020*	0.19	0.717	0.611	-0.03	0.936
**Workload (Watt)**	0.115	0.1	0.261	0.288	-0.04	0.765

* p<0.05.

### HR, VO_2_ and workload at AerT

**Fig 2:**
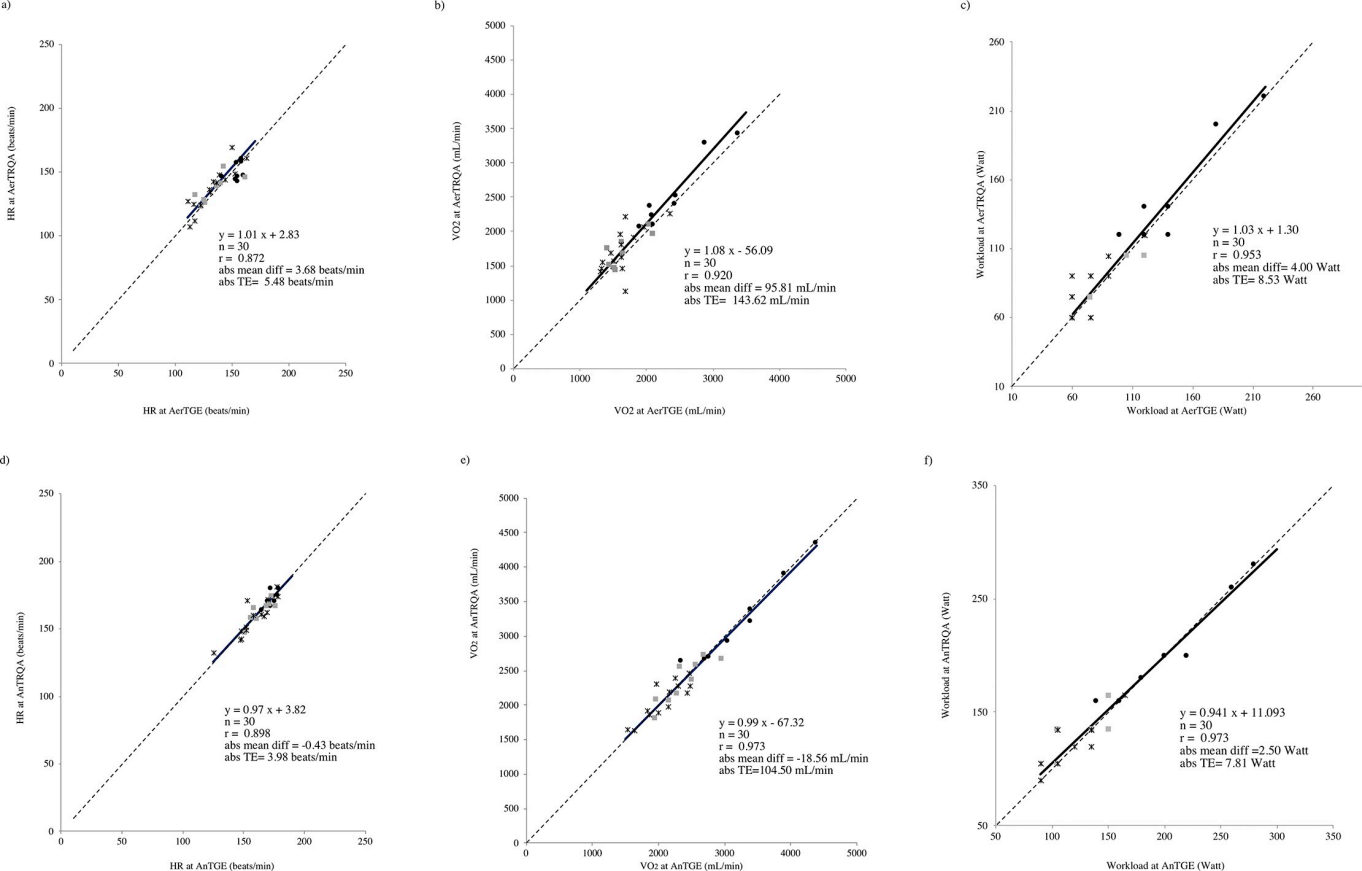
**Regression plots of HR, VO2 and workload (first, second and third line, respectively) estimated by RQA and GE methods at AerT (left) and AnT (right).** Group A (black circle), group B (grey square) and group C (black star).

The agreements between HR, VO_2_ and Workload at the AerT_GE_ and the AerT_RQA_ are presented in Table 4. In all variables (HR, VO_2_ and Workload) OLP regression analysis showed that the AerT_GE_ and the AerT_RQA_ had very strong correlations (*r* >0.8). Slope and intercept values always included the 1 and the 0, respectively. Mean percentage differences were < 6%. HR and VO_2_ at the AerT resulted statistically different between the two evaluation methods (*p* <0.05) while no differences were assessed for Workload at the AerT. The TE for HR and VO_2_ at the AerT was considered acceptable (< 10%), moreover the TE for the Workload at AerT was slightly higher than the acceptable 10% limit [35]. ICC values were excellent (≥ 0.85) for all variables at the AerT [36, 37]. The OLP regression analysis plots of HR, VO_2_ and Workload values at the AerT_GE_ and at the AerT_RQA_ are graphically shown in Fig 2A–2C, respectively. In each graph it is described the OLP regression plot with the linear regression (solid line), the identity (dashed line), the equation, the correlation coefficient (*r*) and the absolute mean differences.

**Table 4:** Agreement between HR, VO_2_ and workload values at AerT and AnT estimated by GE and RQA methods.

Parameter (threshold)	R^2^	Slope	Intercept	Mean diff (%)	*p*	TE (%)	ICC
		(95% CI)	(95% CI)	(95% CI)			(95% CI)
**HR (AerT)**	0.760	1.01	2.83	2.83	0.014*	4.02	0.85
		(0.82 to 1.23)	(-27.74 to 27.87)	(0.80 to 4.87)			(0.68 to 0.93)
**VO**_**2**_ **(AerT)**	0.846	1.08	-56.09	5.59	0.015*	8.40	0.90
		(0.92 to 1.27)	(-404.00 to 240.30)	(1.34 to 9.84)			(0.78 to 0.96)
**Workload (AerT)**	0.908	1.03	1.30	5.26	0.080	10.47	0.95
		(0.91 to 1.17)	(-11.411 to 12.523)	(-0.04 to 10.55)			(0.89 to 0.98)
**HR (AnT)**	0.807	0.97	3.82	-0.18	0.676	2.53	0.90
		(0.81 to 1.16)	(-27.43 to 29.95	(-1.47to 1.10)			(0.80 to 0.95)
**VO**_**2**_ **(AnT)**	0.947	0.99	-67.32	-0.57	0.497	4.67	0.97
		(0.83 to 1.010)	(-54.17 to 399.02)	(2.94 to 1.79)			(0.94 to 0.99)
**Workload (AnT)**	0.947	0.94	11.09	2.82	0.225	6.64	0.97
		(0.86 to 1.03)	(-2.42 to 23.40)	(-0.54 to 6.18)			(0.94 to 0.99)

Determinant coefficient (R^2^), slope and intercept of the regression equations, mean percentage differences (mean diff%), p values, typical percentage error (TE), confidence interval (IC) and intra-class correlation coefficient (ICC).

* p<0.05.

**Fig 3:**
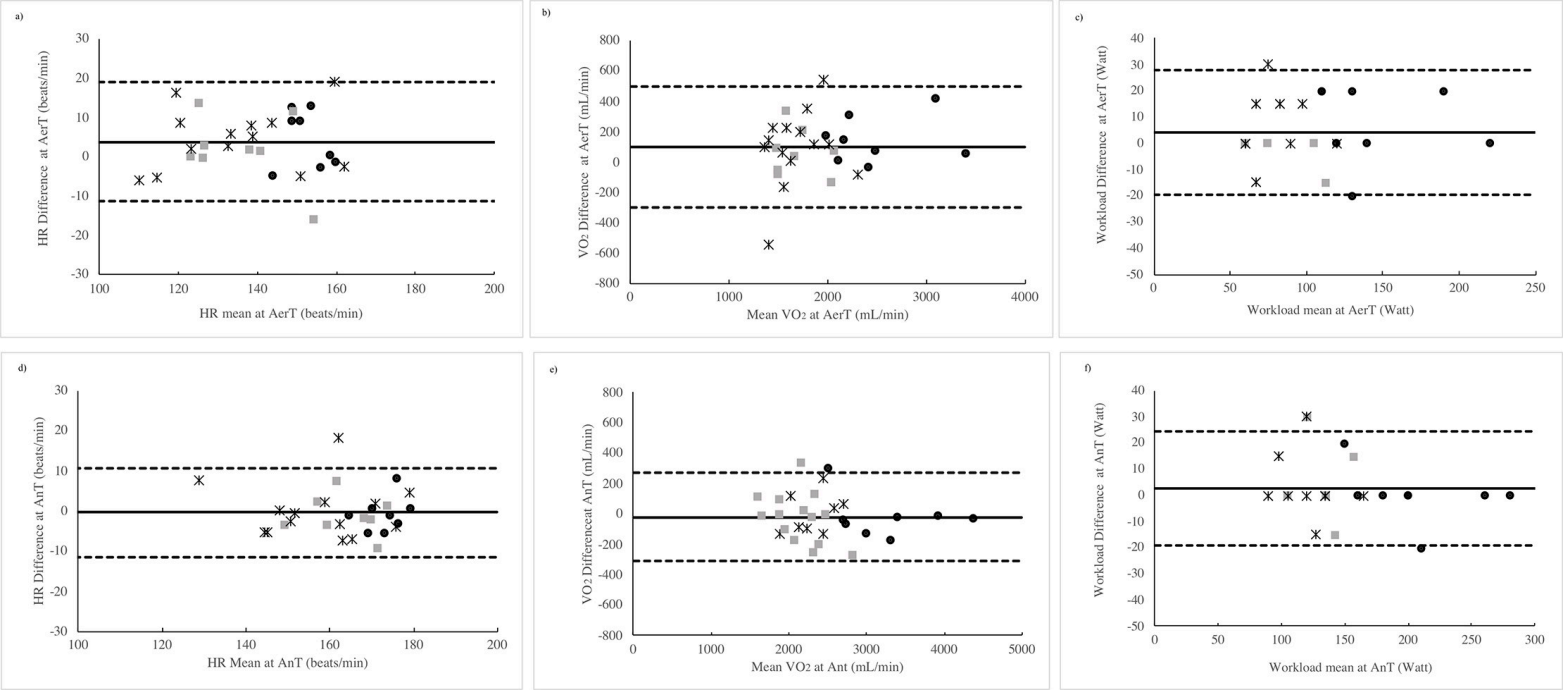
**Bland-Altman plots of HR, VO2 and workload (first, second and third line, respectively) estimated by RQA and GE methods at AerT (left) and AnT (right).** Group A (black circle), group B (grey square) and group C (black star).

For the HR, the VO_2_ and the workload the Bland-Altman plots with the mean difference in the solid line and the 95% LoA in the dashed lines are shown in Fig 3A–3C, respectively.

### HR, VO_2_ and workload at AnT

From Table 2 the value of RER at peak of exercise was higher than the RER at AnT in all three groups and it confirm that exercising to the individual peak capacity allows to measure the AnT in all subjects. The agreement between HR, VO_2_ and Workload at the AnT_GE_ and the AnT_RQA_ are presented in Table 4. In all variables (HR, VO_2_ and Workload) OLP regression analysis showed that the AnT_GE_ and the AnT_RQA_ had very strong correlations (*r* >0.8) [39]. Slope and intercept values always included the 1 and the 0, respectively. Mean percentage differences were <3% and all variables corresponding to the AnT did not result statistically different between the two evaluation methods. For HR, VO_2_ and Workload at the AnT the TE was considered acceptable (<10%) and ICC values were excellent (≥0.90) [36, 38]. The OLP regression analysis plots of HR, VO_2_ and Workload at the AnT and at the AnT are graphically shown in Fig 2D–2F, respectively. For the HR, the VO_2_ and the workload the Bland-Altman plots with the mean difference in the solid line and the 95% LoA are shown in Fig 3D–3F, respectively.

## Discussion

The first aim of the study was to verify if in individuals with different physical fitness levels the new non-linear method based on RQA could detect both ventilatory thresholds. The second aim was to verify the validity of the RQA method compared to the GE method in thresholds detection. To our knowledge, this is the first study assessing the accuracy of the RQA method during the determination of both the AerT and the AnT. In particular, in the present investigation we examined the RQA epoch-by-epoch procedure in order to analyze HRV time series during phase transitions.

Even though for HR and VO_2_ at the AerT significant differences were found between the two methodologies (RQA and GE methods), no differences were detected for Workload at the AerT and for HR, VO_2_ and Workload at the AnT. Differences between RQA and GE methods assessed using effect size statistics (as Cohen’s d) were trivial (<0.2) for all parameters except for HR at AerT (small effect size = 0.24). Furthermore, the absence of methods-by-group interactions in all variables at both the thresholds indicated that the RQA method is not influenced by the level of physical fitness of the subjects. Therefore, this method can be applied in individuals practicing different sports and with different physical fitness levels in situations where gas analysis is not available. These results permitted us to move to the second aim of the study which consisted in verifying the validity of the RQA method against the GE method during thresholds identification through different validation parameters (OLP regression, TE, ICC, and the Bland Altman plots) individually for HR, VO_2_ and Workload variables corresponding at the AerT and the AnT.

Regarding the two methods of AerT detection (AerT_GE_ and AerT_RQA_), the OLP regression in all variables (HR, VO_2_ and Workload) showed very strong correlations [39]. Neither fixed or proportional biases were present in all parameters and mean percentage differences were less than 5% for HR and Workload and less than 6% for VO_2_. As observed in the RM ANOVA results, HR and VO_2_ at the AerT showed significant differences between the two methods, even if the differences were respectively small and trivial. Indeed, both these parameters showed an acceptable TE (HR 4.02%; VO_2_ 8.40%) and excellent ICC values (HR 0.85; VO_2_ 0.90) [36–39]. Therefore, for all these reasons the statistical differences detected between the two methods can be considered not relevant for practical applications. In particular, the low percentage of TE in the heart rate indicated that this variable at the AerT_RQA_ represents the most accurate parameter to be monitored. To conclude, the Workload corresponding to the AerT did not result statistically different between the two evaluation methods and showed in the ICC an excellent value of 0.95. However, this parameter showed a TE slightly higher than the acceptable limit (10.47%) [39].

These first results about the validity of the RQA method for the detection of the AerT could be summarized stating that the AerT_RQA_ is a valid method to use as alternative of gas analysis. In particular, the heart rate at AerT is considered the most valid parameter to monitor using the RQA method, and the determination of the AerT with this approach loses accuracy with VO_2_ and Workload variables. Between the different exercise variables, the HR seems to be not only the most accurate but also the most sustainable, indeed this parameter can be easily recorded during training using non-invasive, not expensive, time-efficient devices which can be applied routinely and simultaneously in a large number of athletes [19]. In the field of exercise assessment, the heart rate variability has been largely used to identify the AerT in several specific populations as diabetic and cardiac patients [40, 41], healthy individuals [42–44], competitive cyclists and triathletes [45], and professional soccer players [46]. However, little is known about the accuracy of the alternative approach of the RQA of HRV time series. Indeed, this method so far has been used only for the identification of the AerT in obese subjects [3]. The present work enlarges the boundaries of this methodology confirming the efficacy of RQA analysis also in individuals with a higher physical fitness level. Therefore, the utilization of the AerT_RQA_ may provide an important training intensity guidance for the identification of the exercise zone which can be covered predominantly by the aerobic metabolism [1, 5, 25].

Even regarding the two methods of AnT detection (AnT_GE_ and AnT_RQA_), the OLP regression in all parameters (HR, VO2 and Workload) showed very strong correlations. [39] and neither fixed or proportional biases were detected. In addition, mean percentage differences were less than 1% for HR and VO_2_ and less than 3% for Workload and no significant differences were found in all variables. Moreover, the acceptable TE values (HR 2.53%; VO_2_ 4.67%, Workload 6.64%) and the excellent ICC values (HR 0.90; VO_2_ 0.97; Workload 0.97) confirmed the validity of all parameters corresponding at the AnT_RQA_ [35–38]. As it was observed in the AerT, the lowest TE found in HR demonstrated that this parameter is the most accurate to use for the detection of the AnT with the RQA method.

Based on the aforementioned findings we believe that the RQA approach is a valid method to assess AnT. In addition, comparing the accuracy of the different variables, the heart rate at the AnT seems to be the most valid and sustainable parameter to use with the RQA method. The HRV has been utilized to estimate the AnT in different exercise evaluation fields [9–11, 41, 45, 46]. However, to our knowledge, this is the first study which attempted to directly examine the non-linear approach of the RQA of HRV time series in the estimation of the AnT. Considering the high importance that the AnT detection has for sport performance [1, 7, 8], these results may provide a useful contribution in training prescription and evaluation.

Comparing the validity of the AerT_RQA_ and AnT_RQA_ (with OLP regression, TE, ICC and the Bland Altman plots) it appeared that the AnT_RQA_ has a better accuracy than AerT_RQA_ and other studies showed similar results [9–11, 45]. This lower accuracy in the AerT_RQA_ may be explained by methodological problems in the determination of this threshold using gas analyzers. Indeed, it seems that the accuracy, the intra- and inter-observer reliability, the repeatability and the percentage of indeterminate thresholds of the AerT_GE_ had been questioned [1, 47]. Therefore, the lower correspondence between AerT_RQA_ and AerT_GE_ may be caused by methodological problems on the GE method in some particular individuals. In view of all these aspects, the use of the RQA method could be used as a supportive approach to gas analysis for all the cases in which the detection of the AerT is ambiguous.

Some limitations may affect our data analysis and have to be mentioned:

- the number of participants of each group is relatively small, although the total sample size was determined by an “*a priori*” power analysis;
- the RQA is a non-linear method in which the correct choice of the parameters is not always trivial and can influence the results [32, 48];
- to synchronize hearth rates and amounts of exchanged gas (to be sure that HR was recorded at the same change of workload), we detected HR values breath-by-breath. Even if, in principle, this procedure could have had an influence on our data since breathing frequency and depth are connected to HRV, we could detect both thresholds similarly to other studies that utilized beat-by-beat HR detection [11, 45, 49];
- our approach was validated using a step protocol, with a workload increase every 60 sec.

Therefore, further investigations including a larger number of subjects, a different sampling of HR (i.e. beat-by-beat) together with a test and re-test procedure, allowing to determine results reliability, are needed. To conclude, future physiological exercise studies should assess if the findings of the present work could be adopted in field tests which do not necessarily require the use of a cycle-ergometer and in other populations different than young, healthy and active individuals.

## Conclusion

The recurrence quantification analysis of heart rate variability time series is a new approach for the determination of ventilatory thresholds in individuals with different physical fitness levels, therefore, it can be used as a valid method for threshold detection. Thus, during an incremental exercise test it is possible to assess sport performance and delineate the intensity of training zones using non-invasive and low-cost devices. Based on the present results, coaches could obtain information about the training trend of their athletes even when gas analysis is non convenient or not possible.

## Supporting information

S1 Table (XLSX)

S2 Table CPET output. (PDF)

S1 File Parameter description. (DOCX)

## Abbreviations

HR: Heart Rate
HRV: Heart Rate Variability
CPET: Cardiopulmonary exercise test
RPE: Rate of Perceived Exertion
RQA: Recurrence Quantification Analysis
GE: Gas Exchange
AerT_GE_: Aerobic Gas Exchange Threshold
AnT_GE_: Anaerobic Gas Exchange Threshold
AerT_RQA_: Aerobic Recurrence Quantification Analysis Threshold
AnT_RQA_: Anaerobic Recurrence Quantification Analysis Threshold
DET: percent of determinism, the percent of recurrence points which form diagonal lines
LAM: percent of laminarity, the percent of recurrence points which form vertical lines
BMI: Body Mass Index
BIA: Bioelectrical Impedance Analysis
FM%: Fat Mass percentage
VO_2_: Volume of Oxygen consumption
VCO_2_: Volume of Carbon dioxide production
VE: ventilation
OLP: Ordinary Least Product
TE: Typical percentage Error
ICC: Intraclass correlation coefficients
IC: confidence interval
RER: Respiratory Exchange Ratio
LoA: Limit of agreement

## References

[1] Meyer T, Lucía A, Earnest CP, Kindermann W. A conceptual framework for performance diagnosis and training prescription from submaximal gas exchange parameters—theory and application. Int J Sports Med. 2005;26 Suppl 1: S38–48. doi: 10.1055/s-2004-830514 15702455

[2] Binder RK, Wonisch M, Corra U, Cohen-Solal A, Vanhees L, Saner H, et al. Methodological approach to the first and second lactate threshold in incremental cardiopulmonary exercise testing. Eur J Cardiovasc Prev Rehabil. 2008, 15(6):726–34 doi: 10.1097/HJR.0b013e328304fed4 19050438

[3] Zimatore G, Gallotta MC, Innocenti L, Bonavolontà V, Ciasca G, De Spirito et al. Recurrence quantification analysis of heart rate variability during continuous incremental exercise test in obese subjects. Chaos. 2020;30(3): 033135. doi: 10.1063/1.5140455 32237785

[4] Emerenziani GP, Gallotta MC, Migliaccio S, Ferrari D, Greco EA, Saavedra FJ et al. Effects of an individualized home-based unsupervised aerobic training on body composition and physiological parameters in obese adults are independent of gender. J Endocrinol Invest. 2018;41(4): 465–473. doi: 10.1007/s40618-017-0771-2 29080964 PMC5852201

[5] Skinner JS, McLellan TH. The transition from aerobic to anaerobic metabolism. Res Q Exerc Sport 1980;51 (1): 234–48. doi: 10.1080/02701367.1980.10609285 7394286

[6] Emerenziani G.P, Migliaccio S., Gallotta M.C, Lenzi A, Baldari C, Guidetti L. Physical exercise intensity prescription to improve health and fitness in overweight and obese subjects: a review of the literature. Health. 2013;5(6A2): 113–121. doi: 10.4236/health.2013.56A2017 24453455

[7] Conconi F, Ferrari M, Ziglio PG, Droghetti P, Codeca L. Determination of the anaerobic threshold by a noninvasive field test in runners. J Appl Physiol Respir Environ Exerc Physiol. 1982;52(4): 869–73. doi: 10.1152/jappl.1982.52.4.869 7085420

[8] Cabo JV, Martinez-Camblor P, Del Valle M. Validity of the modified Conconi test for determining ventilatory threshold during on-water rowing. J Sports Sci Med. 2011;1;10(4): 616–23. ;PMCID: PMC3761509 24149549 PMC3761509

[9] Cassirame J, Tordi N, Fabre N, Duc S, Durand F, Mourot L. Heart rate variability to assess ventilatory threshold in ski-mountaineering. Eur J Sport Sci. 2014;15(7): 615–22. doi: 10.1080/17461391.2014.957729 25228474

[10] Giovanelli N, Scaini S, Billat V, Lazzer S. A new field test to estimate the aerobic and anaerobic thresholds and maximum parameters. Eur J Sport Sci. 2020;20(4): 437–443. doi: 10.1080/17461391.2019.1640289 31267837

[11] Cottin F, Médigue C, Lopes P, Leprêtre PM, Heubert R, Billat V. Ventilatory thresholds assessment from heart rate variability during an incremental exhaustive running test. Int J Sports Med. 2007;28(4): 287–94. doi: 10.1055/s-2006-924355 17024637

[12] Buchheit M, Solano R, Millet GP. Heart-rate deflection point and the second heart-rate variability threshold during running exercise in trained boys. Pediatr Exerc Sci. 2007;19(2): 192–204. doi: 10.1123/pes.19.2.192 17603142

[13] Webber CL Jr, Zbilut JP. Dynamical assessment of physiological systems and states using recurrence plot strategies. J Appl Physiol. 1994;76(2): 965–73. doi: 10.1152/jappl.1994.76.2.965 8175612

[14] Zimatore G, Cavagnaro M. Recurrence Analysis of Otoacoustic Emissions. In Recurrence Quantification Analysis: Theory and Best Practices. 2015;253–278. ISBN: 978-3-319-07154-1

[15] Zimatore G, Cavagnaro M, Skarzynski PH, Fetoni AR, Hatzopoulos S. Detection of Age-Related Hearing Losses (ARHL) via Transient-Evoked Otoacoustic Emissions. Clin Interv Aging. 2020;15:927–935. doi: 10.2147/CIA.S252837 32606634 PMC7319522

[16] Zimatore G, Garilli G, Poscolieri M, Rafanelli C, Terenzio Gizzi F et al. The remarkable coherence between two Italian far away recording stations points to a role of acoustic emissions from crustal rocks for earthquake analysis. Chaos. 2017;27(4): 043101. doi: 10.1063/1.4979351 28456156

[17] Orlando G, Zimatore G. Recurrence quantification analysis of business cycles. Chaos, Solitons and Fractals. 2018;110, 82–94. doi: 10.1016/j.chaos.2018.02.032

[18] Orlando G, Zimatore G. Business cycle modeling between financial crises and black swans: Ornstein-Uhlenbeck stochastic process vs Kaldor deterministic chaotic model. Chaos. 2020;30(8): 083129. doi: 10.1063/5.0015916 32872798

[19] Buchheit M. Monitoring training status with HR measures: do all roads lead to Rome? Front Physiol. 2014;27;5:73. doi: 10.3389/fphys.2014.00027 24578692 PMC3936188

[20] Zakynthinaki MS. Modelling Heart Rate Kinetics. PLOS ONE. 2015;10(4):e0118263. doi: 10.1371/journal.pone.0118263 25876164 PMC4395265

[21] Mongin D, Chabert C, Caparros AU, Guzmán JFV, Hue O, Alvero-Cruz JR, et al. The complex relationship between effort and heart rate: a hint from dynamic analysis. Physiol Meas. 2020;41(10):105003. doi: 10.1088/1361-6579/abbb6e 33164909

[22] Robertson RJ, Goss FL, Dube J, Rutkowski J, Dupain M, Brennan C et al. Validation of the adult OMNI scale of perceived exertion for cycle ergometer exercise. Med Sci Sports Exerc. 2004;36(1): 102–8. doi: 10.1249/01.MSS.0000106169.35222.8B 14707775

[23] Nieman DC, Austin MD, Dew D, Utter AC. Validity of COSMED’s quark CPET mixing chamber system in evaluating energy metabolism during aerobic exercise in healthy male adults. Res Sports Med. 2013;21(2): 136–45. doi: 10.1080/15438627.2012.757227 23541100

[24] Hagan RD, Smith MG. Pulmonary ventilation in relation to oxygen uptake and carbon dioxide production during incremental load work. Int J Sports Med. 1984;5(4): 193–7. doi: 10.1055/s-2008-1025904 6434443

[25] Hollmann W. 42 years ago—development of the concepts of ventilatory and lactate threshold. Sports Med. 2001;31(5): 315–20. doi: 10.2165/00007256-200131050-00002 11347682

[26] Baldari C, Guidetti L. A simple method for individual anaerobic threshold as predictor of max lactate steady state. Med Sci Sports Exerc. 2000;32(10): 1798–802. doi: 10.1097/00005768-200010000-00022 11039656

[27] Trulla L, Giuliani A, Zbilut J P, Webber C L Jr. Recurrence quantification analysis of the logistic equation with transients. Phys Lett A. 1996;223(4): 255–260. 10.1016/S0375-9601(96)00741-4

[28] Marwan N, Romano M C, Thiel M, Kurths J. Recurrence plots for the analysis of complex systems. Phy. Rep. 2007;438(5–6): 237–329

[29] Gorban A N, Smirnova E V, Tyukina T A. Correlations, risk and crisis: From psychology to finance. Physica A. 2010;389(16), 3193–3217. doi: 10.1016/j.physa.2010.03.035

[30] Webber, C. L., see http://homepages.luc.edu/cwebber for RQC.exe and RQE.exe files belong to RQA ver. 8.1 are included in RQA software ver.14.1 (2012) (last accessed 15th January 2020)

[31] Zbilut JP, Thomasson N, Webber CL. Recurrence quantification analysis as a tool for nonlinear exploration of nonstationary cardiac signals. Med Eng Phys. 2002;24(1): 53–60. doi: 10.1016/s1350-4533(01)00112-6 11891140

[32] Marwan N, Zou Y, Wessel N, Riedl M, Kurths J. Estimating coupling directions in the cardiorespiratory system using recurrence properties. Philos Trans A Math Phys Eng Sci. 2013;371(1997): 20110624. doi: 10.1098/rsta.2011.0624 23858487

[33] Guidetti L, Meucci M, Bolletta F, Emerenziani GP, Gallotta MC, Baldari C. Validity, reliability and minimum detectable change of COSMED K5 portable gas exchange system in breath-by-breath mode. PLoS One. 2018;31;13(12): e0209925. doi: 10.1371/journal.pone.0209925 30596748 PMC6312326

[34] Hopkins WG., Marshall SW, Batterham AM, Hanin J. Progressive statistics for studies in sports medicine and exercise science. Medicine & Science in Sports & Exercise. 2009;41(1):3–13. doi: 10.1249/MSS.0b013e31818cb278 19092709

[35] Bland JM, Altman DG. Statistical methods for assessing agreement between two methods of clinical measurement. Lancet. 1986;8;1(8476): 307–10. 10.1016/S0140-6736(86)90837-8 2868172

[36] Alexander D L J, Tropsha A, Winkler D A. Beware of R^2^: Simple, Unambiguous Assessment of the Prediction Accuracy of QSAR and QSPR Models. J. Chem. Inf. Model. 2015;55, 1316–1322. doi: 10.1021/acs.jcim.5b00206 26099013 PMC4530125

[37] Cicchetti D V & Sparrow S A. Developing criteria for establishing interrater reliability of specific items: Applications to assessment of adaptive behavior. American Journal of Mental Deficiency. 1981;86 (2), 127–137. 7315877

[38] Perinetti G. StaTips Part IV: Selection, interpretation and reporting of the intraclass correlation coefficient. South Eur J Orthod Dentofac Res. 2018;5(1): 3–5.

[39] Chan YH. Biostatistic: correlational analysis. Singapore Med J. 2003;44(12): 614–9. 14770254

[40] Sales MM, Campbell CS, Morais PK, Ernesto C, Soares-Caldeira LF, Russo P et al. Noninvasive method to estimate anaerobic threshold in individuals with type 2 diabetes. Diabetol Metab Syndr. 2011;3(1): 1. doi: 10.1186/1758-5996-3-1 21226946 PMC3033241

[41] Mourot L, Tordi N, Bouhaddi M, Teffaha D, Monpere C, Regnard J. Heart rate variability to assess ventilatory thresholds: reliable in cardiac disease? Eur J Prev Cardiol. 2012;19(6): 1272–80. doi: 10.1177/1741826711423115 21914684

[42] Dourado VZ, Banov MC, Marino MC, de Souza VL, Antunes LC, McBurnie MA. A simple approach to assess VT during a field walk test. Int J Sports Med. 2010;31(10): 698–703. doi: 10.1055/s-0030-1255110 20617483

[43] Rogers B, Giles D, Draper N, Hoos O, Gronwald T. A New Detection Method Defining the Aerobic Threshold for Endurance Exercise and Training Prescription Based on Fractal Correlation Properties of Heart Rate Variability. Front Physiol. 2021;11: 596567. doi: 10.3389/fphys.2020.596567 33519504 PMC7845545

[44] Shiraishi Y, Katsumata Y, Sadahiro T, Azuma K, Akita K, Isobe S, et al. Real‐Time Analysis of the Heart Rate Variability During Incremental Exercise for the Detection of the Ventilatory Threshold. Journal of the American Heart Association. 2018;7(1): e006612. doi: 10.1161/JAHA.117.006612 29307865 PMC5778955

[45] Cottin F, Leprêtre PM, Lopes P, Papelier Y, Médigue C, Billat V. Assessment of ventilatory thresholds from heart rate variability in well-trained subjects during cycling. Int J Sports Med. 2006;27(12): 959–67. doi: 10.1055/s-2006-923849 17190003

[46] Naranjo J, De la Cruz B, Sarabia E, De Hoyo M, Domínguez-Cobo S. Heart Rate Variability: a Follow-up in Elite Soccer Players Throughout the Season. Int J Sports Med, 2015; 36(11):881–6. doi: 10.1055/s-0035-1550047 26140687

[47] Zignoli A, Fornasiero A, Rota P, Muollo V, Peyré-Tartaruga L A, Low D A, et al. Oxynet: A collective intelligence that detects ventilatory thresholds in cardiopulmonary exercise tests. Eur J Sport Sci, 2021;1–11. doi: 10.1080/17461391.2020.1866081 33331795

[48] Henriques T, Ribeiro M, Teixeira A, Castro L, Antunes L, Costa-Santos C. Nonlinear Methods Most Applied to Heart-Rate Time Series: A Review. Entropy, 2020;22(3):309. doi: 10.3390/e22030309 33286083 PMC7516766

[49] Merati G, Rampichini S, Cè E, Sangiovanni M, Castiglioni P, Di Rienzo M et al. Ventilatory threshold detection: a new method based on heart rate variability. Computers in Cardiology, 2004, Chicago, IL, USA. 2004; pp. 221–224. doi: 10.1109/CIC.2004.1442912

