# Supplementary material for: Recurrence quantification analysis of heart rate variability to detect both ventilatory thresholds
Source: PLoS One. 2021 Oct 7;16(10):e0249504. doi: 10.1371/journal.pone.0249504 (PMC8496840; doi:10.1371/journal.pone.0249504)
Supplement: S2 Table — (PDF) [file pone.0249504.s002.pdf]

OUTPUT FILE relative to one subject by Quark RMR-CPET Cosmed™, Rome, Italy

First 10 rows of Column 1-15

t	Rf	VT	VE	IV	VO2	VCO2	RQ	O2exp	CO2exp	VE/VO2	VE/VCO2	VO2/Kg	METS	HR
s	1/min	L(btps)	L/min	mL	mL/min	mL/min	---	mL	mL	---	---	mL/min/Kg	---	bpm
00:14	6,42	1,075	6,898	1017	198,8633675	166,4692281	0,84	188,5	32,2	34,7	41,4	2,62	0,7	110
00:16	27,78	0,39	10,833	378	366,4671176	281,5407516	0,77	65,9	12,6	29,6	38,5	4,82	1,4	111
00:17	39,74	0,28	11,126	325	415,7261281	287,5964432	0,69	46,4	9	26,8	38,7	5,47	1,6	114
00:20	22,64	0,914	20,694	826	940,4113678	654,7183064	0,7	143,6	35,7	22	31,6	12,37	3,5	115
00:23	21,13	0,567	11,979	500	423,4736101	292,7884941	0,69	95,2	17,2	28,3	40,9	5,57	1,6	115
00:24	38,71	0,271	10,49	181	416,6225002	274,5580477	0,66	44	8,9	25,2	38,2	5,48	1,6	113
00:26	25,32	0,775	19,62	796	938,7331968	614,606147	0,65	119,8	29,9	20,9	31,9	12,35	3,5	113
00:28	33,71	0,492	16,584	492	727,6037882	469,9582843	0,65	78,3	17,3	22,8	35,3	9,57	2,7	114
00:30	30,3	0,684	20,727	636	966,0008459	612,8016842	0,63	107,2	25	21,5	33,8	12,71	3,6	115
00:32	28,17	0,716	20,169	651	856,5976291	566,8290313	0,66	115,1	24,9	23,5	35,6	11,27	3,2	116

First 10 rows of Columns 28, 29, 30

Grade	Speed	Phase
%	Kmh	---
	3	NONE
	3	EXERCISE
	3	EXERCISE
	3	EXERCISE
	3	EXERCISE
	3	EXERCISE
	3	EXERCISE
	3	EXERCISE
	3	EXERCISE

3 EXERCISE
