# Supplementary material for: Recurrence quantification analysis of heart rate variability to detect both ventilatory thresholds
Source: PLoS One. 2021 Oct 7;16(10):e0249504. doi: 10.1371/journal.pone.0249504 (PMC8496840; doi:10.1371/journal.pone.0249504)
Supplement: S1 File — (DOCX) [file pone.0249504.s003.docx]

**Supporting information**

Group=competitive rowers (A), recreational rowers (B), other recreational sports; Age=years; Sex=male (M), female (F); Height=cm ; Weight=kg; BMI (kg/m²)=body mass index (kg/m²); FM=fat mass expressed as kg; FFM=fat free mass expressed as kg; pFM=fat mass expressed as percentage; pFFM=fat free mass expressed as percentage; VO2 AerT_RQA=oxygen uptake at aerobic recurrence quantification analysis threshold expressed as ml/min; VO2/BW AerT_RQA=oxygen uptake at aerobic recurrence quantification analysis threshold expressed as ml/kg/min; HR AerT_RQA=heart rate at aerobic recurrence quantification analysis threshold; Workload AerT_RQA=workload at aerobic recurrence quantification analysis threshold; RER AerT_RQA=respiratory exchange ratio at aerobic recurrence quantification analysis threshold; VO2 AnT_RQA=oxygen uptake at anaerobic recurrence quantification analysis threshold expressed as ml/min; VO2/BW AnT_RQA=oxygen uptake at anaerobic recurrence quantification analysis threshold expressed as ml/kg/min; HR AnT_RQA=heart rate at anaerobic recurrence quantification analysis threshold; Workload AnT_RQA=workload at anaerobic recurrence quantification analysis threshold; RER AnT_RQA=respiratory exchange ratio at anaerobic recurrence quantification analysis threshold; VO2 AerT_GE=oxygen uptake at aerobic gas exchange threshold expressed as ml/min; VO2/BW AerT_GE=oxygen uptake at aerobic gas exchange threshold expressed as ml/kg/min; HR AerT_GE=heart rate at aerobic gas exchange threshold; Workload AerT_GE=workload at aerobic gas exchange threshold; RER AerT_GE=respiratory exchange ratio at aerobic gas exchange threshold; VO2 AnT_GE=oxygen uptake at anaerobic gas exchange threshold expressed as ml/min; VO2/BW AnT_GE=oxygen uptake at anaerobic gas exchange threshold expressed as ml/kg/min; HR AnT_GE=heart rate at anaerobic gas exchange threshold; Workload AnT_GE=workload at anaerobic gas exchange threshold; RER AnT_GE=respiratory exchange ratio at anaerobic gas exchange threshold; VO2_Peak=oxygen uptake at peak exercise expressed as ml/min; VO2/BW_Peak=oxygen uptake at peak exercise expressed as ml/kg/min; HR_Peak=heart rate at peak exercise; Workload_Peak=workload at peak exercise; RER_Peak=respiratory exchange ratio at peak exercise; RPE_Peak=rate of perceived exertion at peak exercise.
